# Effects of stretching exercises on human gait: a systematic review and meta-analysis

**DOI:** 10.12688/f1000research.25570.2

**Published:** 2020-10-30

**Authors:** Thomas Vialleron, Arnaud Delafontaine, Sebastien Ditcharles, Paul Fourcade, Eric Yiou

**Affiliations:** 1 CIAMS, Univ. Paris-Sud., Université Paris-Saclay, Orsay, 91405, France; 2 CIAMS, Université d'Orléans, Orléans, Orléans, 45067, France; 3 ENKRE, Saint Maurice, Ile de France, 94410, France

**Keywords:** stretching, gait, performance, balance, physical therapy

## Abstract

**Background:** Stretching is commonly used in physical therapy as a rehabilitation tool to improve range of motion and motor function. However, is stretching an efficient method to improve gait, and if so, for which patient category?

**Methods:** A systematic review of randomized and non-randomized controlled trials with meta-analysis was conducted using relevant databases. Every patient category and every type of stretching programs were included without multicomponent programs. Data were meta-analysed where possible. Estimates of effect sizes (reported as standard mean difference (SMD)) with their respective 95% confidence interval (95% CI) were reported for each outcome. The PEDro scale was used for the quality assessment.

**Results:** Twelve studies were included in the analysis. Stretching improved gait performance as assessed by walking speed and stride length only in a study with a frail elderly population, with small effect sizes (both SMD= 0.49; 95% CI: 0.03, 0.96; PEDro score: 3/10). The total distance and the continuous walking distance of the six-minute walking test were also improved only in a study in an elderly population who had symptomatic peripheral artery disease, with large effect sizes (SMD= 1.56; 95% CI: 0.66, 2.45 and SMD= 3.05; 95% CI: 1.86, 4.23, respectively; PEDro score: 5/10). The results were conflicting in healthy older adults or no benefit was found for most of the performance, spatiotemporal, kinetic and angular related variables. Only one study (PEDro score: 6/10) showed improvements in stance phase duration (SMD=-1.92; 95% CI: -3.04, -0.81), swing phase duration (SMD=1.92; 95 CI: 0.81, 3.04), double support phase duration (SMD= -1.69; 95% CI: -2.76, -0.62) and step length (SMD=1.37; 95% CI: 0.36, 2.38) with large effect sizes.

**Conclusions:** There is no strong evidence supporting the beneficial effect of using stretching to improve gait. Further randomized controlled trials are needed to understand the impact of stretching on human gait.

## Introduction

Gait is the medical term used to describe the human whole body movement of walking ^1^. Gait involves internal and external forces that act on the body to move the center of mass (COM) across a given distance ^2^. It depends on many biomechanical features that can be observed during gait analysis such as center of mass shift, joint range of motion (ROM), forces, muscle activity, joint moments, and joint powers ^3^. Spatiotemporal features (e.g. velocity, step length, stride length, step with, step variability) and kinematics parameters (ROM) can be observed subjectively with functional evaluations by clinicians (e.g. the Tinetti test ^4^ or the timed up and go test ^5^), but, it can be further objectified with biomechanical analysis in a laboratory ^2^. Kinetics variables (the forces that cause the body to move) must be collected in a laboratory environment with force plates (e.g. 6– 9 for recent studies that used this technic).

Gait is a highly complex motor skill that is classically considered as an integrative measure and a predictor of health in older adults (e.g. 10; cf. 11 and also 12 for recent research topics on this matter). The loss of gait or its alteration with pathological conditions are known to be related to mortality, especially in the elderly (e.g. 13, 14), stressing the importance of addressing gait disorders in physiotherapy. Gait requires body propulsion and balance control for safe progression, two “subtasks” that require the coordination of multiple skeletal muscles and the integration of sensory information arising from the vestibular, visual and somatosensory systems ^15– 17^. As such, gait may expose populations with sensory or motor deficits to the risk of falling with serious consequences for health and autonomy. For these reasons, improving gait is a major aim in rehabilitation for most neurological/orthopaedic disorders, such as stroke or Parkinson’s disease, and for frail older adults. Various therapeutic methods have been used to improve gait, such as resistance training ^18^, endurance training ^19^, balance training ^20^, whole body vibrations (for a complete review, see Fischer *et al.*, 2019 ^21^), multi-component exercise programs ^22^ and stretching ^23^.

The successful completion of numerous daily life activities is conditioned by the ability to move efficiently through a sufficient ROM ^24^. Recent studies on gait initiation ^25– 27^ and seat-to-stand task ^28, 29^ showed that the experimental restriction of postural chain ROM induced by orthosis wear in young healthy adults led to instability and lower motor performance. It is well established that ROM significantly decreases with aging ^30– 35^ and more generally with reduced functional demand (e.g. sedentarity, immobilization, disease etc.) ^24^. Consequently, stretching has become an important part of many sport and rehabilitation programs to maintain or improve ROM, reduce stiffness and promote physical activity. This method has been applied in older adults ^36, 37^, patients with stroke ^38^, Parkinson’s disease ^39^, multiple sclerosis ^40^, plantar fasciitis ^41^ and spastic paraplegia ^42^, for example. In sport programs, the influence of stretching on motor performance remains an issue of debate, although recent reviews conclude that maximal muscle performance (e.g. force, power, jump height, reaction time, etc.) is impaired primarily immediately after long durations of stretch (>90 seconds) ^43, 44^. To date, no review has collected results on the relationship between stretching and locomotor performance in rehabilitation programs.

Hence, the purpose of this article is to analyse the effects of a stretching program on gait in each patient category by means of a systematic literature review and meta-analysis, comparing the gait outcomes of the intervention groups with the control groups. It will contribute to provide evidence-based practice from scientific data in order to integrate stretching in rehabilitation programs in a reasoned manner.

## Methods

### Design and literature screening

The Preferred Reporting Items for Systematic Reviews and Meta-Analyses (PRISMA) methodology was employed in this systematic review ^45^. A completed PRISMA checklist was submitted to an online repository ( *Reporting guidelines)*.

PubMed, Science Direct, Springer and Sage databases were used for a comprehensive systematic literature search for articles published prior to 28 April 2020 with no time limit. In addition, a manual search was conducted using the reference list of selected studies. The keywords used for the search strategy in PubMed were: “stretching” AND (gait OR walk). We included only articles published in English or French.

The selection procedure was conducted by two experts in rehabilitation (TV and AD). Disagreements were discussed with a third expert in a group until a mutual consensus was reached. First, a review was performed on all available titles obtained from the literature search with the selected keywords. All relevant or potentially relevant titles were included in the subsequent phase. Then, the abstracts were reviewed with all relevant or potential articles included in the following phase. Finally, full-text articles were reviewed to ensure that only relevant studies were included. In the same way, reference lists of all included articles were reviewed to possibly include articles through cross-referencing.

### Inclusion and exclusion criteria

We included randomized controlled trials (RCT) and controlled clinical trials (CCT) published in peer-reviewed journals that aimed to explore the effects of stretching on gait parameters. We included all categories of subjects, all stretching techniques and different durations of treatment since standardized protocols are lacking in the purpose of the present study. Gait could be evaluated by functional tests, electromyographic (EMG) or biomechanical analysis. The following exclusion criteria were used: lack of gait assessment, non-application of muscle stretching, multimodal exercise programs, no control group, case report and review.

### Data extraction and main measurements examined

Data were extracted from the selected articles by one of the authors (TV). The extracted data were checked by another author (AD) and disagreements were resolved with a third (EY).

The following data were extracted for each selected article: (1) the names of the authors and the date of publication; (2) the number of subjects involved in the experiment with their characteristics and breakdown in each group; (3) stretching training details (in the following order: number of participants, stretching technique, muscle groups stretched, number of sets, duration of stretch, frequency, protocol duration); (4) control group details; and (5) the main outcomes related to gait with the main results. When information could not be provided, it was indicated by a “?”.

### Quality and risk of bias assessment

The PEDro scale was used to assess the risk of bias, and thus the methodological quality of the selected studies ^46^. This scale was chosen for its ability to provide an overview of the external (criterion 1), internal (criteria 2–9) and statistical (criteria 9 and 10) validity of clinical trials. The scale is divided into 11 criteria, but the first is not calculated in the total score. The output of each criterion could be either “yes” (y), “no” (n) or “do not know” (?). A “y” was given a score of one point, while an “n” or “?” was assigned zero points. Studies with a total score of 5–10/10 (≥ 50%) were considered to be of high quality, and scores of 0–4/10 (<50%) of low quality ^47^. Two evaluators independently assessed the quality of the included studies. In the event of disagreement, a group discussion was held with a third expert to reach a consensus.

### Statistical analysis

Estimates of effect sizes (comparing the intervention group and the control group) accompanied with a measure of statistical uncertainty (95% confidence interval [95% CI]) were calculated for each outcome when sufficient data were reported. Estimates of effect sizes were reported by standard mean difference (SMD) and their respective 95% CI. In this way, the magnitude of the overall effect can be quantified as trivial (<0.2), small (0.2–0.49), moderate (0.5–0.79) or large (≥0.8) ^48, 49^. When data were lacking to calculate estimates of effect sizes, exact p values were reported.

When at least two studies used the same outcome, meta-analysis was performed, comparing the intervention groups with the control groups. When outcomes were identified in only one study, no meta-analysis could be performed but the effect of intervention was still calculated, reporting the estimate of effect size and its 95% confidence interval. Statistical analysis and figures (i.e. forest plot to facilitate the visualization of values) were produced using a random-effect model in Review Manager software (RevMan, v 5.3, Cochrane Collaboration, Oxford UK). A random-effect model was used to take into account heterogeneity between study effects. Statistical heterogeneity was calculated using the I ^2^ and Cochrane Q statistic tests ^48^. Statistical significance was set at p<0.05.

### Level of evidence

The strength of evidence of primary outcomes was established as described by Van Tulder *et al.* 2003 ^50^ based on effect size estimates with a measure of statistical uncertainty (SMD; 95% CI), statistical heterogeneity (I ^2^) when applicable (multiple studies) and risk of bias (PEDro scale). The level of evidence was considered strong with consistent findings among multiple high-quality RCT (at least two RCT with a PEDro score ≥5/10 that were statistically homogenous: I ^2^ p≥0.05). The level of evidence was considered moderate with consistent findings among multiple low-quality RCT and/or CCT (two trials with a PEDro score <5/10 that were statistically homogenous) and/or one high quality RCT. The level of evidence was considered limited when only one low quality RCT and/or CCT was identified. The level of evidence was conflicting when there was inconsistency among multiple trials (I ^2^ p < 0.05).

## Results

### Included studies

**Figure 1.**
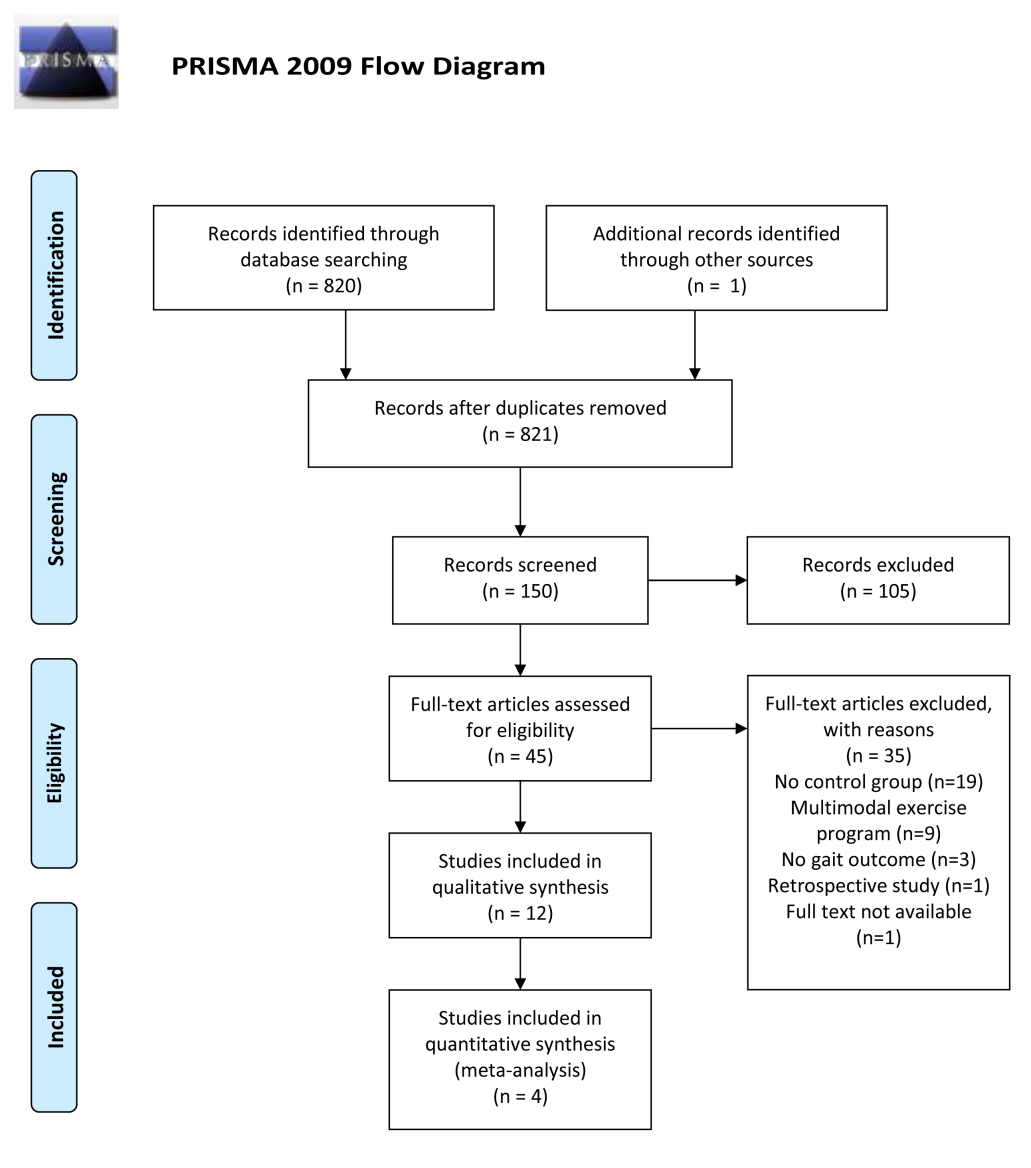
PRISMA flow chart of study selection process.

A total of 821 titles were screened in the first search stage, one more was included through cross-referencing, and 671 were excluded because they did not concern our research question. Following exclusion, 150 studies were considered for an abstract review. A further 105 were excluded in this second stage because they did not meet the inclusion criteria. Finally, 45 full-text articles were assessed for eligibility with 33 not accepted ( Figure 1).

Thus, 12 articles were ultimately included in this systematic review. Six studies evaluated the effects of stretching in healthy older adults ^23, 51– 55^, one in a frail elderly population ^56^, one study in an elderly population with stable symptomatic peripheral artery disease ^57^, one in stroke patients ^58^, one study in adults with limited ankle ROM associated with a history of lower limb overuses injury ^59^, one study in healthy adults with limited ankle dorsiflexion ROM ^60^ and one in healthy young adults ^61^. A summary of the studies selected is provided in Table 1, and their quality assessment is reported in Table 2. The results in different patient categories are reported below.

**Table 1.** Summary of the included studies.

Studies	Population	Stretching group	Control group	Outcomes and main results
Kerrigan *et al.,* 2003 ^53^	96 healthy older adults, ? (≥65 years)	n= 47, static stretching, hip flexors, 4 sets, 30 seconds, twice daily, 10 weeks	n=49, static shoulder deltoid-stretching, same protocol	No significant difference between groups for hip extension (SMD= 0.22; 95% CI: -0.18, 0.62), hip torque (SMD= 0.35; 95% CI: -0.06, 0.75), anterior pelvic tilt (SMD= -0.35 ; 95% CI : -0.76, 0.05), ankle plantar flexion ROM (SMD= -0.05, 95% CI : -0.45, 0.35), ankle plantar flexion power (SMD= 0.00 ; 95% CI : -0.40, 0.40), hip extension (SMD= 0.22 ; 95% CI : -0.19, 0.62) and hip torque (SMD= 0.35; 95% CI: -0.06, 0.75)
Gajdosik *et al.,* 2005 ^23^	19 community dwelling older women, ? (65–89 years)	n=10, static stretching, ankle plantar flexors, 10 sets, 15 seconds, 3 times per week, 8 weeks	n=9, no exercise.	No significant difference between groups for 10MWT (SMD= -0.76; 95% CI: -1.70, 0.18)
Christiansen, 2008 ^51^	40 healthy older adults, 72.10±4.70 years	N= 20, static stretching, hip flexors, 3 sets, 45 seconds, twice daily, 8 weeks	n=20, maintain their current level of physical activity	No significant difference between groups for gait speed (SMD= -0.32; 95% CI: -0.97, 0.33), hip extension (SMD= 0.22; 95% CI: -0.43, 0.86), stride length (SMD= - 0.14 ; 95% CI : -0.79, 0.50), ankle dorsiflexion (SMD= 0.29 ; 95% CI : -0.36, 0.94)
Cristopoliski *et al.,* 2009 ^52^	20 healthy elderly women, 65.90±4.20 years	n=12, static stretching, hip flexors and extensors, ankle plantar flexors, 4 sets, 60 seconds, 3 sessions per week, 12 sessions	n=8, no specific activity in this period	Significant improvement in favor of stretching group for gait speed (SMD= 1.32 ; 95% CI : 0.32, 2.32), anterior pelvic tilt (SMD= -2.52 ; 95% CI : -3.77, -1.27), stand phase duration (SMD= -1.92; 95% CI: -3.04, -0.81), swing phase duration (SMD= 1.92 ; 95% CI : 0.81, 3.04), double support phase duration (SMD= -1.69; 95% CI: -2.76, -0.62), step length (SMD= 1.37 ; 95% CI : 0.36, 2.38) and pelvic rotation (SMD= 1.37 ; 95% CI : 0.36, 2.38) No significant difference between groups for cycle duration (SMD= - 0.24 ; 95% CI : -1.14, 0.66), heel-contact velocity (SMD= -0.46 ; 95% CI : -1.37, 0.45), toe clearance (SMD= 0.91 ; 95% CI : -0.04, 1.86), lateral pelvic tilt (SMD= 0.93 ; 95% CI : -0.02, 1.88) and knee ROM (SMD= 0.23 ; 95% CI : -0.67, 1.12)
Watt *et al.,* 2011 ^55^	82 healthy elderly subjects, 72,6±6 years	n= 43, static stretching, hip flexors, 2 sets, 60 seconds, 2 sessions daily stretching, 10 weeks	N= 39, shoulder abductor static stretching, same protocol	Significant improvement in favor of stretching group for gait speed (SMD= 0.47; 95% CI: 0.03, 0.91) No significant difference between groups for hip extension (SMD= 0.18; 95% CI: -0.25, 0.62), anterior pelvic tilt (SMD=0.07 ; 95% CI : -0.36, 0.51), stride length (SMD= 0.54 ; 95% CI : -0.01, 1.08)
Locks *et al.,* 2012 ^54^	23 healthy older individuals, 67.5±2.12 years	n=10, static stretching, knee extensors, ankle dorsiflexor, knee flexors, ankle plantar flexors, 4 sets, 60 seconds, twice a week ,12 weeks	n=13 a one-hour seminar on healthy living every four weeks and did not perform any physical or therapeutic exercise.	No significant difference between groups for 6MWT (SMD= -0.04; 95% CI : -0.86, 0.79)
Watt *et al.,* 2011 ^56^	74 frail elderly subjects, 77.00±8.00 years	n=33, static stretching, hip flexors, 2 sets, 60 seconds, 2 sessions per day, 10 weeks	n=41, shoulder abductor stretching program, same protocol	No significant difference between groups in peak hip extension, (SMD= 0.22; 95% CI: -0.24, 0.68), anterior pelvic tilt (SMD= -0.05; 95% CI: -0.51, 0.41) and cadence (SMD= 0.13; 95% CI: -0.33, 0.59) Significant improvements in favor of the stretching group in walking speed and stride length (both SMD= 0.49; 95% CI: 0.03, 0.96)
Hotta *et al.,* 2019 ^57^	13 elderly patients with symptomatic peripheral artery disease, ?	n= 13, static stretching, ankle plantar flexor stretching, 1 set, 30 minutes, 5 sessions per week, 4 weeks	n= 13, no stretching intervention (cross- over intervention)	Significant improvements in favor of the stretching group for both total walking distance and continuous walking distance with large effect sizes (SMD= 1.56; 95% CI: 0.66, 2.45 and SMD= 3.05; 95% CI: 1.86, 4.23 respectively)
Kim *et al.,* 2013 ^58^	24 patients with stroke, 53.30±3.16 years	n=12, static stretching, ankle plantar flexors, 1 set, 20 minutes, 4 times a week, 4 times a week, 6 weeks	n= 12, conventional physical therapy as in the stretching group	No significant difference between groups in sway of the center of pressure (SMD=0.75; 95% CI: -0.09, 1.58)
Johanson *et al.,* 2006 ^59^	19 adults with limited passive ankle- dorsiflexion ROM (less than 8 degrees) and a history of lower limb overuse injury, 30.30± 9.80 years	n=11, static stretching, ankle plantar flexors, 5 sets, 30 seconds, 2 times daily, 3 weeks	n= 8, continue all of their usual activities	No significant difference between groups in ankle dorsiflexion during gait in both right and left ankle (SMD= 0.50; 95% CI: -0.42, 1.43 and SMD= 0.41; 95% CI: -0.52, 1.33 respectively) and for time-to-heel-off during the stance phase of gait in both right and left ankle (SMD= -0.50; 95% CI: -1.43, 0.43 and SMD= -0.48; 95% CI: -1.41, 0.45 respectively)
Johanson *et al.,* 2009 ^60^	16 healthy adults with limited passive ankle-dorsiflexion ROM (less than 5 degrees), 27.40±8.20 years	n=8, static stretching, ankle plantar flexors, 4 sets, 30 seconds, 2 times daily, 3 weeks	n=8, no physical activity or stretching programs involving the lower extremities for 3 weeks	No significant difference between groups in ankle dorsiflexion (SMD= 0.53; 95% CI: -0.48, 1.53), maximum knee extension (SMD= -0.07; 95% CI: -1.05, 0.91), medial and lateral gastrocnemius activities (SMD= 0.37; 95% CI: -0.62, 1.36 and SMD= 0.00; 95% CI=: -0.98, 0.98 respectively)
Godges *et al.,* 1993 ^61^	16 healthy, athletic, male college students, 21.00±l.00 years	n=9, static stretching, hip flexors, 3 sets, 2 minutes, 2 sessions per week, 3 weeks	n=7, continue their current activity levels	No significant difference between groups in gait economy (SMD= 0.83; 95% CI: -0.21, 1.87)

SMD: standard mean difference, CI: confidence interval, 10MWT: 10-meter walk test, 6MWT: 6-minute walk test, ROM: ROM, ?: information not provided.

**Table 2.** Quality assessment of the included studies.

Study	Eligibility criteria specified	Random allocation	Concealed allocation	Groups similar at baseline	Participant blinding	Therapist blinding	Assessor blinding	<15% dropouts	Intention- to-treat analysis	Between- group difference reported	Point estimate and variability reported	Total score	Patient category
Kerrigan *et al.,* 2003	y	y	n	y	n	n	y	y	n	y	y	6	Healthy older adults
Gajdosik *et al.,* 2005	y	y	y	y	n	n	n	y	y	y	y	7	
Christiansen, 2008	y	y	n	y	n	n	n	y	n	y	y	5	
Cristopoliski *et al.,* 2009	y	y	n	y	n	n	n	y	y	y	y	6	
Watt *et al.,* 2011	y	y	n	n	n	n	y	n	n	n	y	3	
Locks *et al.,* 2012	y	n	n	y	n	n	n	n	n	y	y	3	
Watt *et al.,* 2011	y	y	n	?	n	n	y	n	n	n	y	3	Frail older adults
Hotta *et al.,* 2009	y	y	n	y	n	n	n	y	n	y	y	5	Peripheral artery disease
Kim *et al.,* 2013	n	n	n	y	n	n	n	n	n	y	y	3	Stroke
Johanson *et al.,* 2006	y	y	n	n	n	n	n	y	y	y	y	5	Lower limb overuse injury
Johanson *et al.,* 2009	y	y	n	y	n	n	n	y	y	y	y	6	Limited ankle ROM
Godges *et al.,* 1993	y	y	n	?	n	n	n	y	y	y	y	5	Healthy adults

n: criterion not fulfilled; y: criterion fulfilled; ?: criterion not mentioned; total score: each item (except the first) contributes 1 point to the total score, yielding a PEDro scale score that can range from 0 to 10.

### Results in different patient categories

***Healthy older adults***

**Description of the studies and quality assessment**

Six studies examined the effects of stretching on healthy elderly subjects ^23, 51– 55^. Regarding the characteristics of the subjects, the average sample size was 46.6±33.9 subjects (ranging from 19 ^23^ to 96 subjects ^53^) and the mean age was 70.1±3.6 years (ranging from 65.40 ^52^ to 75.30 years ^23^). Regarding the characteristics of the training programs, the average training duration was 8.6±2.7 weeks (ranging from 4 ^52^ to 12 weeks ^54^), with an average frequency of 8.3±6.2 sessions per week (ranging from 2 ^54^ to 14 sessions ^51, 53, 55^). The average number of sets per session was 4.5±2.8 sets (ranging from 2 ^55^ to 10 sets ^23^), with an average stretching time of 45.0±18.9 seconds (ranging from 15 ^23^ to 60 seconds ^52, 54, 55^). Static stretching was provided in all studies. The muscle groups stretched were the hip flexors ^51– 55^, ankle plantar flexors ^23, 51, 52, 54^, ankle dorsiflexors ^54^, hip extensors ^52^, knee extensors and flexors ^54^. There was great heterogeneity in gait outcomes. Angular variables during gait included peak hip extension ^51, 53, 55^, ankle plantar flexion during gait ^53^, ankle ROM during gait ^52^, anterior pelvis tilt ^52, 55^, knee ROM, pelvic rotation, lateral pelvic tilt and hip ROM ^52^. Spatiotemporal variables were: gait speed ^51, 52, 55^, stance and swing duration, double support phases, step length ^52^ and stride length ^52, 55^. Kinetic variables were hip torque and ankle plantar flexion power ^53^. Finally, two functional tests were used: the 10-meter walk test (10MWT) ^23^ and the 6-minute walk test (6MWT) ^54^. Regarding the quality of the studies, the average PEDro score was 4.6±1.6 and one study was identified as a non-randomized trial ^54^. The range of score varied from 3 ^54, 55^ to 7 ^23^.

**Meta-analyses**

**Figure 2.**
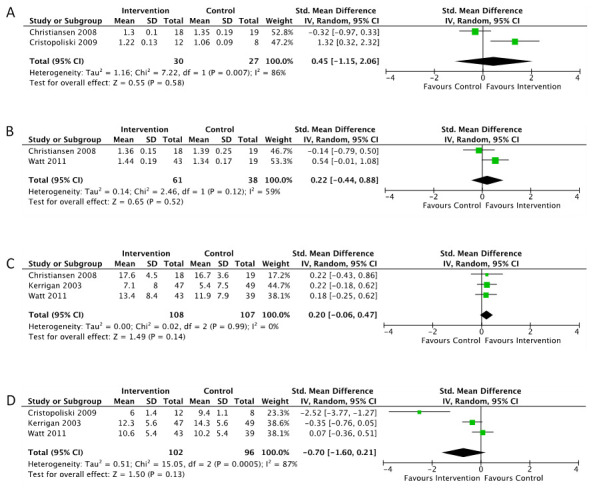
Comparisons between intervention and control groups for gait speed ( **A**), stride length ( **B**), hip extension ( **C**) and anterior pelvic tilt ( **D**) in healthy older adults.

Four meta-analysis were conducted for the following outcomes ( Figure 2): gait speed, stride length, hip extension during gait and anterior pelvic tilt.

*Gait speed:* For gait speed ( Figure 2A), two studies were included in the meta-analysis ^51, 52^. One study was excluded because intervention and control groups were not similar at baseline ^55^. Statistical analysis showed no significant difference between groups (SMD= 0.45; 95% CI: -1.15, 2.06), with heterogeneous results (I ^2^=86%, p=0.007). Thus, the level of evidence was conflicting.

*Stride length:* For stride length ( Figure 2B), two studies were included in the meta-analysis ^51, 55^. Statistical analysis showed no significant difference between groups (SMD= 0.22; 95% CI: -0.44, 0.88), with consistent results (I ^2^=59%, p=0.12). Only one study was of high quality ^51^, thus a moderate level of evidence supports the lack of beneficial effect of stretching to improve stride length in the elderly.

*Hip extension:* For hip extension during gait (kinematic data) ( Figure 2C), three studies were included in the meta-analysis ^51, 53, 55^. Statistical analysis showed no significant difference between groups (SMD= 0.20; 95% CI: -0.06, 0.47), with consistent results (I ^2^=0%, p=0.99). Two studies were of high quality ^51, 53^, thus a strong level of evidence supports the lack of beneficial effect of stretching to improve hip ROM during gait in the elderly.

*Anterior pelvic tilt:* For anterior pelvic tilt ( Figure 2D), three studies were included in the meta-analysis ^52, 53, 55^. Statistical analysis showed no significant difference between groups (SMD= -0.70; 95% CI: -1.60, 0.21), with heterogeneous results (I ^2^=87%, p<0.01). Thus, the level of evidence was conflicting.

**Effects of interventions in other outcomes**

For the outcomes below, no meta-analysis could be performed because only one study was identified. Nevertheless, for each outcome, effect size estimates with a measure of statistical uncertainty (95% CI) were provided.

*Angular variables during gait initiation:* The study of Christiansen *et al.* (2008) showed no significant difference between stretching and control groups for ankle dorsiflexion during gait (SMD=0.29; 95% CI: -0.36, 0.94) with a moderate level of confidence (PEDro score: 5/10). The study of Kerrigan *et al.* (2003) showed no significant difference between groups for ankle plantar flexion (SMD=-0.05; 95% CI: -0.45, 0.35), with a moderate level of confidence (PEDro score: 6/10). The study of Cristopoliski *et al.* (2009) showed no significant difference between groups for lateral pelvic tilt (SMD= 0.93; 95% CI: -0.02, 1.88) and knee ROM (SMD= 0.23; 95% CI: -0.67, 1.12), with a moderate level of confidence (PEDro score: 6/10).

*Kinetic variables:* The study of Kerrigan *et al.* (2003) showed no significant difference between groups for hip torque (SMD= 0.35; 95% CI: -0.06, 0.75) and ankle plantar flexion power (SMD=0.00; 95% CI: -0.40, 0.40), with a moderate level of confidence (PEDro score: 6/10).

*Spatiotemporal variables:* The study of Cristopoliski *et al.* (2009) showed no significant difference between groups for cycle duration (SMD= -0.24; 95% CI: -1.14, 0.66), heel contact velocity (SMD= -0.46; 95% CI: -1.37, 0.45) and toe clearance (SMD= 0.91; 95% CI: -0.04, 1.86). However, the study showed significant decreases with large effect sizes in stance phase duration (SMD=-1.92; 95% CI: -3.04, -0.81), double support phase duration (SMD= -1.69; 95% CI: -2.76, -0.62) in favour of the stretching group as compared to the control group. Additionally, the authors found significant increases with large effect sizes of swing phase duration (SMD=1.92; 95 CI: 0.81, 3.04) and step length (SMD=1.37; 95% CI: 0.36, 2.38) in favour of the stretching group as compared to the control group. The study obtained a PEDro score of 6/10, thus, the level of evidence for these outcomes was moderate.

*Functional tests:* The study of Gajdosik *et al.* (2005) showed no significant difference between groups for the 10MWT (SMD= -0.76; 95% CI= -1.70, 0.18), with a moderate level of confidence (PEDro score: 7/10). The study of Locks *et al.* (2012) showed no significant improvement of the 6MWT in favour of the stretching group as compared to the control group (SMD= -0.04; 95% CI:-0.86, 0.79) with a limited level of confidence (low quality CCT with a PEDro score of 3/10).

***Frail elderly***

**Description of the study and quality assessment**

The study of Watt *et al.* 2011 examined the effects of stretching on frail elderly subjects ^56^. Regarding the characteristics of the subjects, 74 subjects were included, and the mean age was 77.0±8.0 years. Regarding the characteristics of the training programs, the stretching program lasted ten weeks, with a frequency of 14 sessions per week (two sessions per day). Participants performed two sets per session, holding the stretch for 60 seconds (static stretching), alternating the right and left limb (four minutes in total). The muscle group stretched was the hip flexors. The outcomes were cadence (steps/minute), walking speed (meters/second), stride length (meters) peak hip extension (degree) and peak anterior pelvic tilt (degree). Regarding the quality assessment, the study was identified as RCT and had an average PEDro score of 3 (low level of evidence).

**Effects of intervention**

The study of Watt *et al.* (2011) showed no significant difference between groups in angular variables, i.e. peak hip extension and anterior pelvic tilt (SMD= 0.22; 95% CI: -0.24, 0.68 and SMD= -0.05; 95% CI: -0.51, 0.41 respectively). There was also no significant difference for cadence (SMD= 0.13; 95% CI: -0.33, 0.59). However, the study showed significant improvements in favour of the stretching group with small effect sizes in some performance-related variables, i.e. walking speed and stride length (both SMD= 0.49; 95% CI: 0.03, 0.96).

***Elderly with symptomatic peripheral artery disease***

**Description of the study and quality assessment**

The study of Hotta *et al.* (2019) examined the effects of stretching in elderly with symptomatic peripheral artery disease ^57^. Regarding the characteristics of the subjects, 13 subjects were included and the mean age was not mentioned. Regarding the characteristics of the training programs, the stretching program lasted four weeks, with a frequency of five sessions per week. Participants performed one set daily, holding the stretch for 30 minutes (static stretching with splints). The muscle group stretched was ankle plantar flexors. The gait outcome was 6MWT. Regarding the quality assessment, the study was identified as RCT and had an average PEDro score of 5 (moderate level of evidence).

**Effects of intervention**

The study of Hotta *et al.* (2019) showed significant improvements in favour of the stretching group for both total walking distance and continuous walking distance with large effect sizes (SMD= 1.56; 95% CI: 0.66, 2.45 and SMD= 3.05; 95% CI: 1.86, 4.23 respectively).

***Stroke***

**Description of the study and quality assessment**

The study of Kim *et al.* (2013) examined the effects of stretching on stroke patients ^58^. Only a static muscle stretching training group and control group were included in the analysis. Regarding the characteristics of the subjects, 24 subjects were included, and the mean age was 53.3±3.1 years. Regarding the characteristics of the training programs, the stretching program lasted six weeks, with a frequency of four sessions per week. Participants performed one set per session, holding the stretch for 20 minutes (static stretching). The muscle group stretched was ankle plantar flexors. The outcome was the sway of the centre of pressure during the stance phase. Regarding the quality assessment, the study was identified as CCT and had an average PEDro score of 3 (low level of evidence).

**Effects of intervention**

The study of Kim *et al.* (2013) showed no significant difference between groups in the sway of the centre of pressure (SMD=0.75; 95% CI: -0.09, 1.58).

***Young adults with limited ankle ROM and a history of lower limb overuse injury***

**Description of the study and quality assessment**

The study of Johanson *et al.* (2006) examined the effects of stretching on healthy adults with limited passive ankle-dorsiflexion ROM (less than eight degrees) and a history of lower limb overuse injury ^59^. Regarding the characteristics of the subjects, 19 subjects were included and the mean age was 30.3±9.8 years. Regarding the characteristics of the training programs, the stretching program lasted three weeks, with a frequency of two sessions per day. Participants performed five sets per session, holding the stretch for 30 seconds (static stretching). The muscle group stretched was ankle plantar flexors. The outcomes were ankle dorsiflexion and time-to-heel-off during the stance phase of gait. Regarding the quality assessment, the study was identified as RCT and had an average PEDro score of 5 (moderate level of evidence).

**Effects of intervention**

The study of Johanson *et al.* (2006) showed no significant difference between groups in ankle dorsiflexion during gait in both the right and left ankle (SMD= 0.50; 95% CI: -0.42, 1.43 and SMD= 0.41; 95% CI: -0.52, 1.33 respectively). There was also no significant difference between groups for time-to-heel-off during the stance phase of gait in both the right and left ankle (SMD= -0.50; 95% CI: -1.43, 0.43 and SMD= -0.48; 95% CI: -1.41, 0.45 respectively).

***Young adults with limited ankle ROM***

**Description of the study and quality assessment**

The study of Johanson *et al.* (2009) examined the effects of stretching on young adults with limited passive ankle-dorsiflexion ROM (less than 5 degrees) ^60^. It is worth noticed that these participants were not the same than in the study of Johanson *et al.* (2006). In contrast, the characteristics of the training programs were the same as in Johanson *et al.* (2006). In the current study, 16 subjects were included, and the mean age was 27.4±8.2 years. The muscle group stretched was the ankle plantar flexors. The outcomes were maximum ankle dorsiflexion, maximum knee extension and EMG amplitude of the gastrocnemius during the stance phase of gait. Regarding the quality assessment, the study was identified as RCT and had an average PEDro score of 6 (moderate level of evidence).

**Effects of intervention**

The study of Johanson *et al.* (2009) showed no significant difference between groups in angular variables during gait, i.e. maximum ankle dorsiflexion and maximum knee extension (SMD= 0.53; 95% CI: -0.48, 1.53 and SMD= -0.07; 95% CI: -1.05, 0.91 respectively). There was also no significant difference between groups for EMG variables, i.e. medial and lateral gastrocnemius activity (SMD= 0.37; 95% CI: -0.62, 1.36 and SMD= 0.00; 95% CI=: -0.98, 0.98 respectively).

***Healthy young adults***

**Description of the study and quality assessment**

The study of Godges *et al.* (1993) examined the effects of stretching on healthy young adults ^61^. Only a static hip extension stretching group and control group were included in the analysis. Regarding the characteristics of the subjects, 16 subjects were included, and the mean age was 21.0±1.0 years. Regarding the characteristics of the training programs, the stretching program lasted three weeks, with a frequency of two sessions per week. Participants performed three sets per session, holding the stretch for two minutes (static stretching). The muscle group stretched was the hip flexors. The outcome was walking economy (ml/kg/min). Regarding the quality assessment, the study was identified as RCT and had an average PEDro score of 5 (moderate level of evidence).

**Effects of intervention**

The study of Godges *et al.* (1993) showed no significant difference between groups in gait economy in terms of oxygen consumption (SMD= 0.83; 95% CI: -0.21, 1.87).

## Discussion

The aim of this systematic review was to determine the effects of a stretching program on human gait by means of a systematic literature review and meta-analysis. Twelves studies were identified in six different patient categories. Statistical analyses showed no strong level of evidence supporting the beneficial effect of a stretching program to improve any gait outcome. The major issue in conducting meta-analyses and establishing strong level of evidences was the great heterogeneity in gait variables. The results obtained in the different patient categories are discussed in detail below.

### Healthy older adults

The healthy older adult population was the most studied. Two muscle groups were systematically stretched in the six identified studies: hip flexors ^23, 51– 53, 55^ and ankle plantar flexors ^23, 51– 53^. Hip flexor stiffness, associated with reduced hip extension during gait has been demonstrated in the elderly and may alter gait ^62, 63^. In the same way, decreased calf muscle length associated with restricted dorsiflexion ROM is well documented in older adults ^35, 64^. A decreased ankle dorsiflexion ROM has been correlated with poorer balance test scores in the elderly ^65^ and may contribute to an increased risk of falls ^66^. All the studies included in the present analysis showed that specific stretching programs were efficient to improve passive ROM of the targeted joints, but results are more heterogeneous regarding gait performance and dynamic ROM. This led to inconsistency in the results or the impossibility to conclude with a strong level of evidence that a stretching program improves gait in healthy older adults. Moreover, when improvement in ROM or gait performance occurred, it was not associated with a significant increase in dynamic hip extension or ankle dorsiflexion. Only trends toward increased dynamic ROM after stretching interventions were observed ^51, 53, 55^. This observation was consistent in young adults.

When data were meta-analyzed, we ensured that the groups and the training characteristics were similar to limit the risk of bias. This explains that a limited number of studies was included in the meta-analysis. It is worth noticed that the stretching technic was the same (i.e. static stretching), but that details of interventions varied across these studies. For example, both studies selected for the meta-analysis of gait speed included hip flexors and plantar flexors stretching, but, one study included hip extensor stretching ^52^ whereas the other did not ^51^. This difference may partially explain the heterogenous results in the meta-analysis (I ^2^=86%, p=0.007). In the same way, the heterogenous results observed in the meta-analysis of anterior pelvic tilt (I ^2^=87%, <0.01) may be explained by the stretching of additional muscle groups (hip extensors and plantar flexors) in the study of Cristopoliski *et al*. (2009) compared to the other studies (in which only hip flexors were stretched) ^53, 55^. Nevertheless, heterogeneity in the results was not systematically observed between studies that used slight different protocols, as showed by the consistent results int the meta-analyses of stride length (I ^2^=59%, p=0.12) and hip extension (I ^2^=0%, p=0.99). Thus, we assume that we have limited the risk of bias in the meta-analyses.

### Stroke patients

In stroke patients, ankle plantar flexor stretching has been successfully used to improve ankle stiffness ^67– 70^. Decreased plantar flexors stiffness may have a beneficial effect on postural control during gait because *triceps surae* is known to play an important role during gait ^71– 73^ and an increase in muscle stiffness might alter synergistic muscle activities during human gait. However, only one non-randomized study ^58^ was identified and included in the current systematic review. Other studies that used stretching in multicomponent programs ^74– 76^ or in control groups ^77, 78^ were identified but excluded because of the addition of resistance training or the lack of a control group. Nevertheless, it should be noted that some studies showed improvements between pre- and post-stretching conditions. Forrester *et al.* (2014) showed that both robotic ankle mobilizations and manual ankle stretching improved gait velocity in stroke patients at hospital discharge compared to baseline. Similarly, Park *et al.* (2018) showed that both static ankle stretching and ankle mobilizations improved gait speed after four weeks of treatment compared to baseline. Other authors showed that one week of immobilization in dorsiflexed position (casting) followed by one week of plantar flexor stretching and gait training improved gait performances in 10MWT and 6MWT ^74^. Hence, these encouraging results suggest that further randomized controlled trials of good quality are needed to explore the ability of ankle stretching to improve gait parameters in stroke or in other neurological diseases exposing patients to joint stiffness, e.g. Parkinson’s disease ^79^.

### Young adults

In healthy adults, the interest of practicing stretching to improve gait seems limited as they are assumed to have sufficient mobility for walking. Moreover, the included study involved athletic males ^61^, a population that is known to be more flexible than inactive persons ^80^. Stretching should be more indicated when ROM is limited ^24^. However, even in young adults with limited ankle ROM, stretching did not improve dynamic dorsiflexion during gait ^59, 60^. Stretching programs in apparently healthy adults should be more indicated after a prolonged period of reduced functional demand (e.g. immobilization, sedentarity), when ROM is insufficient to practice a specific activity or when high levels of flexibility are required for sport performance (e.g. gymnastics or dance) and in sports that involve stretch-shortening cycles (e.g. basketball, volleyball) ^24^.

### Limitations of the study

Some patient categories were not included in the present review, although muscle stretching is commonly indicated in their clinical care to reduce spasticity ^81^. This is for example the case for children with cerebral palsy ^82^. In fact, we were able to identify studies in the literature focusing on the effects of stretching on gait in this population during the first phase of the present review, but the protocol of these studies combined stretching with another form of training (e.g. 83, 84) or there was no control group (e.g. 85). These studies therefore did not fit with the inclusion criteria of the present systematic review and were consequently excluded. Now, it should be stressed that the effectiveness of static stretching to improve motor function in children with cerebral palsy is still controversial ^86^, although some authors showed that functional stretching exercises may be effective to improve gait ^85^. Further randomized controlled trials are needed to explore the impact of stretching on gait in this population.

To reduce the risk of bias, data were meta-analyzed when at least 2 studies with similar populations and training characteristics were found. Considering these constraints and the great heterogeneity in the gait outcomes, we only performed meta-analyses in healthy older adults for 4 outcomes. The Cochrane Qualitative and Implementation Methods Group recommends the application of Grades of Recommendation, Assessment, Development, and Evaluation (GRADE) in the Evidence from Qualitative Reviews to assess the level of confidence in systematic review with meta-analyses ^87^. However, the GRADE necessitates assessing the risk of publication bias with a funnel plot, determining its asymmetry, which can be performed with at least 10 studies ^88^. In the present study, the meta-analyses included less that 10 randomized controlled trials, so, we chose to implement other guidelines described by a Cochrane collaboration group to assess the level of evidence ^50^. Because this method includes fewer criteria, our confidence in the results must be taken with caution

## Conclusion

Twelve studies were identified, involving a total of 442 subjects. Despite some improvements in isolated studies, statistical analyses showed no strong level of evidence supporting the beneficial effect of using stretching alone to improve gait outcomes in rehabilitation programs. The major obstacle in conducting meta-analyses and establishing strong levels of evidence were the great heterogeneity in gait variables and the low quality of the included studies. Because the effects of stretching are not clear, further randomized controlled trials of good quality are needed to understand the impact of stretching on human gait. Currently, stretching is more recommended to maintain and improve ROM rather than improve gait parameters and should be integrated in multicomponent programs.

## Data availability

### Underlying data

All data underlying the results are available as part of the article and no additional source data are required.

### Reporting guidelines

Harvard Dataverse: PRISMA checklist and PRISMA flow diagram for ‘Effects of stretching exercises on human gait: a systematic review and meta-analysis’, https://doi.org/10.7910/DVN/N8ZXNB ^89^.

Data are available under the terms of the Creative Commons Zero “No rights reserved” data waiver (CC0 1.0 Public domain dedication).

## References

[1] Beauchet O Allali G Sekhon H: Guidelines for Assessment of Gait and Reference Values for Spatiotemporal Gait Parameters in Older Adults: The Biomathics and Canadian Gait Consortiums Initiative. *Front Hum Neurosci.* 2017;11:353. 10.3389/fnhum.2017.00353 28824393 PMC5540886

[2] Dicharry J: Kinematics and kinetics of gait: from lab to clinic. *Clin Sports Med.* 2010;29(3):347–364. 10.1016/j.csm.2010.03.013 20610026

[3] Ounpuu S: The biomechanics of walking and running. *Clin Sports Med.* 1994;13(4):843–863. 10.1016/S0278-5919(20)30289-1 7805110

[4] Tinetti ME: Performance-oriented assessment of mobility problems in elderly patients. *J Am Geriatr Soc.* 1986;34(2):119–126. 10.1111/j.1532-5415.1986.tb05480.x 3944402

[5] Podsiadlo D Richardson S: The Timed “Up & Go”: A Test of Basic Functional Mobility for Frail Elderly Persons. *J Am Geriatr Soc.* 1991;39(2):142–148. 10.1111/j.1532-5415.1991.tb01616.x 1991946

[6] Yiou E Artico R Teyssedre CA: Anticipatory Postural Control of Stability during Gait Initiation Over Obstacles of Different Height and Distance Made Under Reaction-Time and Self-Initiated Instructions. *Front Hum Neurosci.* 2016;10:449. 10.3389/fnhum.2016.00449 27656138 PMC5013047

[7] Yiou E Fourcade P Artico R: Influence of temporal pressure constraint on the biomechanical organization of gait initiation made with or without an obstacle to clear. *Exp Brain Res.* 2016;234(6):1363–1375. 10.1007/s00221-015-4319-4 25990822

[8] Yiou E Teyssèdre C Artico R: Comparison of base of support size during gait initiation using force-plate and motion-capture system: A Bland and Altman analysis. *J Biomech.* 2016;49(16):4168–4172. 10.1016/j.jbiomech.2016.11.008 27855983

[9] Artico R Fourcade P Teyssedre CA: Influence of swing foot strike pattern on balance control mechanisms during gait initiation over an obstacle to clear. In: *Progress in Motor Control*, Amsterdam, Netherlands. 7–10 Jul 2019.;2019. Reference Source

[10] LeBrasseur NK: Gait as an Integrative Measure and Predictor of Health Across Species. *J Gerontol A Biol Sci Med Sci.* 2019;74(9):1411–1412. 10.1093/gerona/glz121 31074770

[11] Blumen HM Cavallari P Mourey F: Editorial: Adaptive Gait and Postural Control: from Physiological to Pathological Mechanisms, Towards Prevention and Rehabilitation. *Front Aging Neurosci.* 2020;12:45. 10.3389/fnagi.2020.00045 32161535 PMC7052351

[12] Yiou E Hamaoui A Allali G: Editorial: The Contribution of Postural Adjustments to Body Balance and Motor Performance. *Front Hum Neurosci.* 2018;12:487. 10.3389/fnhum.2018.00487 30568587 PMC6290066

[13] White DK Neogi T Nevitt MC: Trajectories of Gait Speed Predict Mortality in Well-Functioning Older Adults: The Health, Aging and Body Composition Study. *J Gerontol A Biol Sci Med Sci.* 2013;68(4):456–464. 10.1093/gerona/gls197 23051974 PMC3593620

[14] Rodríguez-Molinero A Herrero-Larrea A Miñarro A: The spatial parameters of gait and their association with falls, functional decline and death in older adults: a prospective study. *Sci Rep.* 2019;9(1):8813. 10.1038/s41598-019-45113-2 31217471 PMC6584504

[15] Allum JH Bloem BR Carpenter MG: Proprioceptive control of posture: a review of new concepts. *Gait Posture.* 1998;8(3):214–242. 10.1016/s0966-6362(98)00027-7 10200410

[16] Horak FB: Postural orientation and equilibrium: what do we need to know about neural control of balance to prevent falls? *Age Ageing.* 2006;35 Suppl 2:ii7–ii11. 10.1093/ageing/afl077 16926210

[17] Mirelman A Shema S Maidan I: Gait. *Handb Clin Neurol.* 2018;159:119–134. 10.1016/B978-0-444-63916-5.00007-0 30482309

[18] Arcolin I Pisano F Delconte C: Intensive cycle ergometer training improves gait speed and endurance in patients with Parkinson’s disease: A comparison with treadmill training. *Restor Neurol Neurosci.* 2015;34(1):125–138. 10.3233/RNN-150506 26684265

[19] Lustosa LP Silva JP Coelho FM: Impact of resistance exercise program on functional capacity and muscular strength of knee extensor in pre-frail community-dwelling older women: a randomized crossover trial. *Rev Bras Fisioter.* 2011;15(4):318–324. 10.1590/S1413-35552011000400010 21971726

[20] Halvarsson A Franzén E Ståhle A: Balance training with multi-task exercises improves fall-related self-efficacy, gait, balance performance and physical function in older adults with osteoporosis: a randomized controlled trial. *Clin Rehabil.* 2015;29(4):365–375. 10.1177/0269215514544983 25142277

[21] Fischer M Vialleron T Laffaye G: Long-Term Effects of Whole-Body Vibration on Human Gait: A Systematic Review and Meta-Analysis. *Front Neurol.* 2019;10:627. 10.3389/fneur.2019.00627 31316447 PMC6611385

[22] Freiberger E Häberle L Spirduso WW: Long-term effects of three multicomponent exercise interventions on physical performance and fall-related psychological outcomes in community-dwelling older adults: a randomized controlled trial. *J Am Geriatr Soc.* 2012;60(3):437–446. 10.1111/j.1532-5415.2011.03859.x 22324753

[23] Gajdosik RL Vander Linden DW McNair PJ: Effects of an eight-week stretching program on the passive-elastic properties and function of the calf muscles of older women. *Clin Biomech (Bristol, Avon).* 2005;20(9):973–983. 10.1016/j.clinbiomech.2005.05.011 16054737

[24] Portero P McNair P: Les étirements musculo-tendineux : des données scientifiques à une pratique raisonnée. *Kinésithérapie, la Revue.* 2015;15(164–165):32–40. 10.1016/j.kine.2015.06.009

[25] Delafontaine A Gagey O Colnaghi S: Rigid Ankle Foot Orthosis Deteriorates Mediolateral Balance Control and Vertical Braking during Gait Initiation. *Front Hum Neurosci.* 2017;11:214. 10.3389/fnhum.2017.00214 28503144 PMC5408009

[26] Delafontaine A Fourcade P Honeine JL: Postural adaptations to unilateral knee joint hypomobility induced by orthosis wear during gait initiation. *Sci Rep.* 2018;8(1):830. 10.1038/s41598-018-19151-1 29339773 PMC5770397

[27] Delafontaine A Honeine JL Do MC: Comparative gait initiation kinematics between simulated unilateral and bilateral ankle hypomobility: Does bilateral constraint improve speed performance? *Neurosci Lett.* 2015;603:55–59. 10.1016/j.neulet.2015.07.016 26197055

[28] Alamini-Rodrigues C Hamaoui A: Effect of three different lumbar splints on posturokinetic capacity when performing the sit-to-stand task. *Ann Phys Rehabil Med.* 2017;60(6):406–409. 10.1016/j.rehab.2016.09.003 27773478

[29] Hamaoui A Alamini-Rodrigues C: Influence of Cervical Spine Mobility on the Focal and Postural Components of the Sit-to-Stand Task. *Front Hum Neurosci.* 2017;11:129. 10.3389/fnhum.2017.00129 28400724 PMC5368949

[30] Grimston SK Nigg BM Hanley DA: Differences in ankle joint complex range of motion as a function of age. *Foot Ankle.* 1993;14(4):215–222. 10.1177/107110079301400407 8359768

[31] James B Parker AW: Active and passive mobility of lower limb joints in elderly men and women. *Am J Phys Med Rehabil.* 1989;68(4):162–167. 10.1097/00002060-198908000-00002 2765206

[32] McKay MJ Baldwin JN Ferreira P: Normative reference values for strength and flexibility of 1,000 children and adults. *Neurology.* 2017;88(1):36–43. 10.1212/WNL.0000000000003466 27881628 PMC5200854

[33] Roach KE Miles TP: Normal hip and knee active range of motion: the relationship to age. *Phys Ther.* 1991;71(9):656–665. 10.1093/ptj/71.9.656 1881956

[34] Soucie JM Wang C Forsyth A: Range of motion measurements: reference values and a database for comparison studies. *Haemophilia.* 2011;17(3):500–507. 10.1111/j.1365-2516.2010.02399.x 21070485

[35] Vandervoort AA Chesworth BM Cunningham DA: Age and sex effects on mobility of the human ankle. *J Gerontol.* 1992;47(1):M17–21. 10.1093/geronj/47.1.m17 1730848

[36] Lee PG Jackson EA Richardson CR: Exercise Prescriptions in Older Adults. * Am Fam Physician.* 2017;95(7):425–432. 28409595

[37] Mora JC Valencia WM: Exercise and Older Adults. * Clin Geriatr Med.* 2018;34(1):145–162. 10.1016/j.cger.2017.08.007 29129214

[38] Beyaert C Vasa R Frykberg GE: Gait post-stroke: Pathophysiology and rehabilitation strategies. *Neurophysiol Clin.* 2015;45(4–5):335–355. 10.1016/j.neucli.2015.09.005 26547547

[39] Abbruzzese G Marchese R Avanzino L: Rehabilitation for Parkinson’s disease: Current outlook and future challenges. *Parkinsonism Relat Disord.* 2016;22 Suppl 1:S60–S64. 10.1016/j.parkreldis.2015.09.005 26360239

[40] Bisht B Darling WG White EC: Effects of a multimodal intervention on gait and balance of subjects with progressive multiple sclerosis: a prospective longitudinal pilot study. *Degener Neurol Neuromuscul Dis.* 2017;7:79–93. 10.2147/DNND.S128872 30050380 PMC6053103

[41] Thong-On S Bovonsunthonchai S Vachalathiti R: Effects of Strengthening and Stretching Exercises on the Temporospatial Gait Parameters in Patients With Plantar Fasciitis: A Randomized Controlled Trial. *Ann Rehabil Med.* 2019;43(6):662–676. 10.5535/arm.2019.43.6.662 31918529 PMC6960082

[42] van Lith BJH den Boer J van de Warrenburg BPC: Functional effects of botulinum toxin type A in the hip adductors and subsequent stretching in patients with hereditary spastic paraplegia. *J Rehabil Med.* 2019;51(6):434–441. 10.2340/16501977-2556 30968942

[43] Behm DG Chaouachi A: A review of the acute effects of static and dynamic stretching on performance. *Eur J Appl Physiol.* 2011;111(11):2633–2651. 10.1007/s00421-011-1879-2 21373870

[44] Kay AD Blazevich AJ: Effect of acute static stretch on maximal muscle performance: a systematic review. *Med Sci Sports Exerc.* 2012;44(1):154–164. 10.1249/MSS.0b013e318225cb27 21659901

[45] Moher D Liberati A Tetzlaff J: Preferred Reporting Items for Systematic Reviews and Meta-Analyses: The PRISMA Statement. * PLoS Med.* 2009;6(7):e1000097. 10.1371/journal.pmed.1000097 19621072 PMC2707599

[46] de Morton NA: The PEDro scale is a valid measure of the methodological quality of clinical trials: a demographic study. * Aust J Physiother.* 2009;55(2):129–133. 10.1016/s0004-9514(09)70043-1 19463084

[47] Lindberg J Carlsson J: The effects of whole-body vibration training on gait and walking ability - A systematic review comparing two quality indexes. * Physiother Theory Pract.* 2012;28(7):485–98. 10.3109/09593985.2011.641670 22214345

[48] Higgins JP Green S: Cochrane Handbook for Systematic Reviews of Interventions. Accessed March 26, 2019. Reference Source

[49] Cohen J: Statistical Power Analysis for the Behavioral Sciences. 2nd ed. L. Erlbaum Associates;1988. 10.4324/9780203771587

[50] van Tulder M Furlan A Bombardier C: Updated Method Guidelines for Systematic Reviews in the Cochrane Collaboration Back Review Group. *Spine (Phila Pa 1976).* 2003;28(12):1290–1299. 10.1097/01.BRS.0000065484.95996.AF 12811274

[51] Christiansen CL: The effects of hip and ankle stretching on gait function of older people. *Arch Phys Med Rehabil.* 2008;89(8):1421–1428. 10.1016/j.apmr.2007.12.043 18674977

[52] Cristopoliski F Barela JA Leite N: Stretching Exercise Program Improves Gait in the Elderly. *Gerontology.* 2009;55(6):614–620. 10.1159/000235863 19713691

[53] Kerrigan DC Xenopoulos-Oddsson A Sullivan MJ: Effect of a hip flexor-stretching program on gait in the elderly. * Arch Phys Med Rehabil.* 2003;84(1):1–6. 10.1053/apmr.2003.50056 12589613

[54] Locks RR Costa TC Koppe S: Effects of strength and flexibility training on functional performance of healthy older people. * Rev Bras Fisioter.* 2012;16(3):184–190. 10.1590/s1413-35552012000300003 22801513

[55] Watt JR Jackson K Franz JR: Effect of a Supervised Hip Flexor Stretching Program on Gait in Elderly Individuals. * PM R.* 2011;3(4):324–329. 10.1016/j.pmrj.2010.11.012 21497318

[56] Watt JR Jackson K Franz JR: Effect of a Supervised Hip Flexor Stretching Program on Gait in Frail Elderly Patients. *PM R.* 2011;3(4):330–335. 10.1016/j.pmrj.2011.01.006 21497319

[57] Hotta K Batchelor WB Graven J: Daily Passive Muscle Stretching Improves Flow-Mediated Dilation of Popliteal Artery and 6-minute Walk Test in Elderly Patients with Stable Symptomatic Peripheral Artery Disease. *Cardiovasc Revasc Med.* 2019;20(8):642–648. 10.1016/j.carrev.2019.05.003 31171470 PMC6698217

[58] Kim TH Yoon JS Lee JH: The Effect of Ankle Joint Muscle Strengthening Training and Static Muscle Stretching Training on Stroke Patients’ C.O.P Sway Amplitude. *J Phys Ther Sci.* 2013;25(12):1613–1616. 10.1589/jpts.25.1613 24409032 PMC3885851

[59] Johanson MA Wooden M Catlin PA: Effects of gastrocnemius stretching on ankle dorsiflexion and time-to heel-off during the stance phase of gait. *Phys Ther Sport.* 2006;7(2):93–100. 10.1016/j.ptsp.2006.02.002

[60] Johanson MA Cuda BJ Koontz JE: Effect of stretching on ankle and knee angles and gastrocnemius activity during the stance phase of gait. *J Sport Rehabil.* 2009;18(4):521–534. 10.1123/jsr.18.4.521 20108853

[61] Godges JJ MacRae PG Engelke KA: Effects of Exercise on Hip Range of Motion, Trunk Muscle Performance, and Gait Economy. *Phys Ther.* 1993;73(7):468–477. 10.1093/ptj/73.7.468 8316580

[62] Kerrigan DC Lee LW Collins JJ: Reduced hip extension during walking: healthy elderly and fallers versus young adults. *Arch Phys Med Rehabil.* 2001;82(1):26–30. 10.1053/apmr.2001.18584 11239282

[63] Kerrigan DC Todd MK Della Croce U: Biomechanical gait alterations independent of speed in the healthy elderly: evidence for specific limiting impairments. *Arch Phys Med Rehabil.* 1998;79(3):317–322. 10.1016/s0003-9993(98)90013-2 9523785

[64] Gajdosik RL Linden VWD Vander Linden DW: Influence of Age on Length and Passive Elastic Stiffness Characteristics of the Calf Muscle-Tendon Unit of Women. *Phys Ther.* 1999;79(9):827–838. 10.1093/ptj/79.9.827 10479783

[65] Mecagni C Smith JP Roberts KE: Balance and ankle range of motion in community-dwelling women aged 64 to 87 years: a correlational study. *Phys Ther.* 2000;80(10):1004–1011. 10.1093/ptj/80.10.1004 11002436

[66] Gehlsen GM Whaley MH: Falls in the elderly: Part II Balance, strength, and flexibility. *Arch Phys Med Rehabil.* 1990;71(10):739–741. 2403279

[67] Gao F Ren Y Roth EJ: Effects of repeated ankle stretching on calf muscle–tendon and ankle biomechanical properties in stroke survivors. *Clin Biomech (Bristol, Avon).* 2011;26(5):516–522. 10.1016/j.clinbiomech.2010.12.003 21211873 PMC3085098

[68] Yeh CY Tsai KH Chen JJ: Effects of prolonged muscle stretching with constant torque or constant angle on hypertonic calf muscles. *Arch Phys Med Rehabil.* 2005;86(2):235–241. 10.1016/j.apmr.2004.03.032 15706549

[69] Yeh CY Chen JJJ Tsai KH: Quantitative analysis of ankle hypertonia after prolonged stretch in subjects with stroke. *J Neurosci Methods.* 2004;137(2):305–314. 10.1016/j.jneumeth.2004.03.001 15262075

[70] Bressel E McNair PJ: The effect of prolonged static and cyclic stretching on ankle joint stiffness, torque relaxation, and gait in people with stroke. *Phys Ther.* 2002;82(9):880–887. 10.1093/ptj/82.9.880 12201802

[71] Honeine JL Schieppati M Gagey O: By counteracting gravity, triceps surae sets both kinematics and kinetics of gait. *Physiol Rep.* 2014;2(2):e00229. 10.1002/phy2.229 24744898 PMC3966244

[72] Honeine JL Schieppati M Gagey O: The Functional Role of the Triceps Surae Muscle during Human Locomotion. *PLoS One.* 2013;8(1):e52943. 10.1371/journal.pone.0052943 23341916 PMC3547017

[73] Neptune RR Kautz SA Zajac FE: Contributions of the individual ankle plantar flexors to support, forward progression and swing initiation during walking. *J Biomech.* 2001;34(11):1387–1398. 10.1016/s0021-9290(01)00105-1 11672713

[74] Carda S Invernizzi M Baricich A: Casting, taping or stretching after botulinum toxin type A for spastic equinus foot: a single-blind randomized trial on adult stroke patients. *Clin Rehabil.* 2011;25(12):1119–1127. 10.1177/0269215511405080 21729974

[75] Ghasemi E Khademi-Kalantari K Khalkhali-Zavieh M: The effect of functional stretching exercises on functional outcomes in spastic stroke patients: A randomized controlled clinical trial. *J Bodyw Mov Ther.* 2018;22(4):1004–1012. 10.1016/j.jbmt.2017.09.021 30368324

[76] Moore SA Jakovljevic DG Ford GA: Exercise Induces Peripheral Muscle But Not Cardiac Adaptations After Stroke: A Randomized Controlled Pilot Trial. *Arch Phys Med Rehabil.* 2016;97(4):596–603. 10.1016/j.apmr.2015.12.018 26763949 PMC5813708

[77] Forrester LW Roy A Krywonis A: Modular Ankle Robotics Training in Early Sub-Acute Stroke: A Randomized Controlled Pilot Study. *Neurorehabil Neural Repair.* 2014;28(7):678–687. 10.1177/1545968314521004 24515923 PMC4127380

[78] Park D Lee JH Kang TW: Four-week training involving ankle mobilization with movement versus static muscle stretching in patients with chronic stroke: a randomized controlled trial. *Top Stroke Rehabil.* 2019;26(2):81–6. 10.1080/10749357.2018.1550614 30477417

[79] Raza C Anjum R Shakeel NUA: Parkinson’s disease: Mechanisms, translational models and management strategies. *Life Sci.* 2019;226:77–90. 10.1016/j.lfs.2019.03.057 30980848

[80] Haff G Triplett NT, National Strength & Conditioning Association (U.S.), eds. Essentials of Strength Training and Conditioning. Fourth edition. Human Kinetics;2016. Reference Source

[81] Smania N Picelli A Munari D: Rehabilitation procedures in the management of spasticity. *Eur J Phys Rehabil Med.* 2010;46(3):423–438. 20927008

[82] Pin T Dyke P Chan M: The effectiveness of passive stretching in children with cerebral palsy. *Dev Med Child Neurol.* 2006;48(10):855–862. 10.1017/S0012162206001836 16978468

[83] Fosdahl MA Jahnsen R Kvalheim K: Effect of a Combined Stretching and Strength Training Program on Gait Function in Children with Cerebral Palsy, GMFCS Level I & II: A Randomized Controlled Trial. *Medicina (Kaunas).* 2019;55(6):250. 10.3390/medicina55060250 31174397 PMC6630432

[84] Kalkman BM Holmes G Bar-On L: Resistance Training Combined With Stretching Increases Tendon Stiffness and Is More Effective Than Stretching Alone in Children With Cerebral Palsy: A Randomized Controlled Trial. *Front Pediatr.* 2019;7:333. 10.3389/fped.2019.00333 31456995 PMC6700382

[85] Elshafey MA Abd-Elaziem A Gouda RE: Functional stretching exercise submitted for spastic diplegic children: a randomized control study. *Rehabil Res Pract.* 2014;2014:814279. 10.1155/2014/814279 25143834 PMC4131100

[86] Kalkman BM Bar-On L O’Brien TD: Stretching Interventions in Children With Cerebral Palsy: Why Are They Ineffective in Improving Muscle Function and How Can We Better Their Outcome? *Front Physiol.* 2020;11:131. 10.3389/fphys.2020.00131 32153428 PMC7047287

[87] Noyes J Booth A Flemming K: Cochrane Qualitative and Implementation Methods Group guidance series-paper 3: methods for assessing methodological limitations, data extraction and synthesis, and confidence in synthesized qualitative findings. *J Clin Epidemiol.* 2018;97:49–58. 10.1016/j.jclinepi.2017.06.020 29247700

[88] Higgins J Green S: Cochrane Handbook for Systematic Reviews of Interventions Version 5.1.0 [Updated March 2011]. The Cochrane Collaboration,2011. Reference Source

[89] Delafontaine A: Effects of stretching exercises on human gait: A systematic review and meta-analysis.Harvard Dataverse, V1.2020. 10.7910/DVN/N8ZXNB PMC7919610 33728043

