# Supplementary material for: Effects of stretching exercises on human gait: a systematic review and meta-analysis
Source: F1000Res. 2020 Oct 30;9:984. Originally published 2020 Aug 13. [Version 2] doi: 10.12688/f1000research.25570.2 (PMC7919610; doi:10.12688/f1000research.25570.2)
Supplement: Peer review history

# Peer review: Reviewer response for version 2

**DOI:** 10.5256/f1000research.30366.r79222

**Published:** 2021-02-26

**Authors:** Maria Garcia Escudero

**Affiliations:** 1 School of Medicine and Health Sciencies, Universidad Católica de Valencia San Vicente Mártir, Valencia, Spain

I believe that the idea of this research study was necessary to carry it out because of the need to clarify stretch treatments and clear protocols to improve the progress of patients.

 I think the methodology used is appropriate although after reviewing the results it is concluded that further randomized controlled trials of good quality are necessary even to offer this line of research to carry out new studies and improve the cohort of patients by categories and sample number.

Are the rationale for, and objectives of, the Systematic Review clearly stated?

Yes

Is the statistical analysis and its interpretation appropriate?

Yes

Are sufficient details of the methods and analysis provided to allow replication by others?

Yes

Are the conclusions drawn adequately supported by the results presented in the review?

Yes

Reviewer Expertise:

NA

I confirm that I have read this submission and believe that I have an appropriate level of expertise to confirm that it is of an acceptable scientific standard.

# Peer review: Reviewer response for version 2

**DOI:** 10.5256/f1000research.30366.r74039

**Published:** 2020-11-09

**Authors:** Fabrice Mégrot

**Affiliations:** 1 Unité Clinique d’Analyse de la Marche et du Mouvement, Centre de Médecine Physique et de Réadaptation pour Enfants Bois-Larris, Croix-rouge française, Lamorlaye, France; 2 BioMécanique et BioIngénierie (BMBI) - UMR 7338, Université de Technologie de Compiègne, Sorbonnes Université, Compiègne, France

Thanks for the corrections. That works.

Are the rationale for, and objectives of, the Systematic Review clearly stated?

Yes

Is the statistical analysis and its interpretation appropriate?

Yes

Are sufficient details of the methods and analysis provided to allow replication by others?

Yes

Are the conclusions drawn adequately supported by the results presented in the review?

Partly

Reviewer Expertise:

motor control, biomechanics, neurosciences, nonlinear dynamics, clinical gait/movement analysis and gross motor function of children with cerebral palsy

I confirm that I have read this submission and believe that I have an appropriate level of expertise to confirm that it is of an acceptable scientific standard.

# Peer review: Reviewer response for version 1

**DOI:** 10.5256/f1000research.28219.r69875

**Published:** 2020-10-09

**Authors:** Xu Wei, Guoning Zhang

**Affiliations:** 1 Department of Orthopaedic Surgery, Tongren Hospital, Shanghai Jiao Tong University School of Medicine, Shanghai, China

1. The purpose of this article is to analyse the effects of a stretching program on gait in each patient category by means of a systematic literature review and meta-analysis, comparing the gait outcomes of the intervention groups with the control groups. This is a very interesting research direction and it has good innovation. But different ages and diseases have different gait results and different stretching training modes and intervention methods. How to eliminate the bias caused by these differences in meta-analysis and comparison?
2. Four meta-analysis were conducted for the following outcomes (Figure 2): gait speed, stride length, hip extension during gait and anterior pelvic tilt. Why choose these four dimensions? Is there any theoretical basis?
3. Kinetic variables: The study of Kerrigan et al. (2003)44 showed no significant difference between groups for hip torque (SMD= 0.35; 95% CI: -0.06, 0.75) and ankle plantar flexion power (SMD=0.00; 95% CI: -0.40, 0.40), with a moderate level of confidence (PEDro score: 6/10).: It seems that gait analysis cannot directly measure muscle power. sEMG can only evaluate muscle recruitment signals to evaluate muscle fiber contraction. Therefore, how is ankle flexion power measured by gait analysis?
4. Twelve studies were included in the analysis. Stretching improved gait performance as assessed by walking speed and stride length only in a study with a frail elderly population, with small effect sizes. There is no strong evidence supporting the beneficial effect of using stretching to improve gait. I think that since stretching can improve gait parameters for adults with special weakness, the effect of stretching on gait is significant, although it has little effect on healthy adults or young adults. Should it be explained? In general, this study made a meta-analysis on the effect of stretching on gait, which has good innovation. However, whether gait analysis has guiding significance for different groups of rehabilitation training is still controversial especially for healthy adults, so the research conclusion of this paper has practical significance in clinical guidance.

Are the rationale for, and objectives of, the Systematic Review clearly stated?

Yes

Is the statistical analysis and its interpretation appropriate?

Partly

Are sufficient details of the methods and analysis provided to allow replication by others?

Yes

Are the conclusions drawn adequately supported by the results presented in the review?

Partly

Reviewer Expertise:

Sports medicine; osteoporosis; osteosarcoma; Spine surgery; Scoliosis

We confirm that we have read this submission and believe that we have an appropriate level of expertise to confirm that it is of an acceptable scientific standard, however we have significant reservations, as outlined above.

# Response

**Published:** 2020-10-20

**Authors:** ARNAUD DELAFONTAINE

**Affiliations:** INRA Université Paris Sud, France

Dear Reviewer

We greatly you for scrutinizing our manuscript and for his relevant comments. We feel that the manuscript has improved.

Comment 1. Different ages and diseases have different gait results and different stretching training modes and intervention methods. How to eliminate the bias caused by these differences in meta-analysis and comparison?

**Reply:** We add some precisions in the discussion section (healthy older adults paragraph) to specify how we have limited the risk of bias:

Please see L353-366: When data were meta-analyzed, we ensured that the groups and the training characteristics were similar to limit the risk of bias. This explains that a limited number of studies was included in the meta-analysis. It is worth noticing that the stretching technic was the same (i.e. static stretching), but that details of interventions varied across these studies. For example, both studies selected for the meta-analysis of gait speed included hip flexors and plantar flexors stretching, but, one study included hip extensor stretching ^52^ whereas the other did not ^51^. This difference may partially explain the heterogenous results in the meta-analysis (I ^2^=86%, p=0.007). In the same way, the heterogenous results observed in the meta-analysis of anterior pelvic tilt (I ^2^=87%, p<0.01) may be explained by the stretching of additional muscle groups (hip extensors and plantar flexors) in the study of Cristopoliski et al. (2009) compared to the other studies (in which only hip flexors were stretched) ^53,55^. Nevertheless, heterogeneity in the results was not systematically observed between studies that used slight different protocols, as showed by the consistent results in the meta-analyses of stride length (I ^2^=59%, p=0.12) and hip extension (I ^2^=0%, p=0.99). Thus, we assume that we have limited the risk of bias in the meta-analyses.

Comment 2. Four meta-analysis were conducted for the following outcomes (Figure 2): gait speed, stride length, hip extension during gait and anterior pelvic tilt. Why choose these four dimensions? Is there any theoretical basis?

**Reply:** Only these four outcomes fitted with the inclusion criteria for meta-analysis. Meta-analyses were performed only when more than one trial was identified for each outcome. Additionally, to reduce the risk of bias, we ensured that the groups were similar. For example, in the study of Watt et al. (2011), the intervention group had a significantly higher gait speed than the control group, so the trial was excluded of the meta-analysis.

Comment 3. Kinetic variables: The study of Kerrigan et al. (2003)44 showed no significant difference between groups for hip torque (SMD= 0.35; 95% CI: -0.06, 0.75) and ankle plantar flexion power (SMD=0.00; 95% CI: -0.40, 0.40), with a moderate level of confidence (PEDro score: 6/10).: It seems that gait analysis cannot directly measure muscle power. sEMG can only evaluate muscle recruitment signals to evaluate muscle fiber contraction. Therefore, how is ankle flexion power measured by gait analysis?

**Reply:** In the study of Kerrigan et al. (2003), “joint torque and power calculations were based on the mass and inertial characteristics of each lower-extremity segment, the derived linear and angular velocities and accelerations of each lower-extremity segment, and the ground reaction force and joint center position estimates. Joint torques and powers were normalized for body weight and height and were reported as external in newton meters per kilogram meters (N.m/kg.m) and watts per kilogram meters (W/kg.m), respectively”.

Unless special recommendation of the reviewer or the Editor, we feel it is not necessary to add how the ankle flexion power was measured in the study of Kerrigan et al. (2003).

Comment 4. I think that since stretching can improve gait parameters for adults with special weakness, the effect of stretching on gait is significant, although it has little effect on healthy adults or young adults. Should it be explained? 

**Reply:** We add some precisions in the discussion section (young adults paragraph) to specify why stretching is less interesting to improve gait parameters in young healthy adults:

Please see L387-390: In healthy adults, the interest of practicing stretching to improve gait seems limited as they are assumed to have sufficient mobility for walking. Moreover, the included study involved athletic males ^61^, a population that is known to be more flexible than inactive persons ^81^. Stretching should be more indicated when range of motion is limited ^24^.

Best regards 

Arnaud Delafontaine 

on behalf of all the authors

# Peer review: Reviewer response for version 1

**DOI:** 10.5256/f1000research.28219.r69874

**Published:** 2020-09-14

**Authors:** Fabrice Mégrot

**Affiliations:** 1 Unité Clinique d’Analyse de la Marche et du Mouvement, Centre de Médecine Physique et de Réadaptation pour Enfants Bois-Larris, Croix-rouge française, Lamorlaye, France; 2 BioMécanique et BioIngénierie (BMBI) - UMR 7338, Université de Technologie de Compiègne, Sorbonnes Université, Compiègne, France

Authors propose a systematic review and meta-analysis to investigate the effects of stretching programs on gait and to determine how stretching might be valuable for rehabilitation. From 150 studies identified through systematic searches and critical appraisal of the literature, 12 articles were considered by the authors.

 The various demographics of the participants in the reported studies implied a mixed range of age and aetiology, even if old persons, either with an asymptomatic or a pathological health status, were the most represented individuals.

 In young adults, two studies by Johanson are reported. Is it not clear if both groups of young adults with limited range of motion were identical in both studies and had the same characteristics? If yes, merging the two studies should make the deal, if not please provide specification about the second group.

 Overall, the methodology is correct, and the meta-analysis well described. The results show weak effects of stretching on gait parameters.

 The main issue arises from the various parameters which are considered in all the studies, both during gait initiation and straight walking (spatiotemporal parameters, kinematics, joint strength and dynamics, muscle activity, etc…). Therefore, in the results and the discussion, grouping the types of parameters according to their role in the process of walking would add value to this manuscript. The effects, demonstrated or not, have not all the same meaning depending on whether functional parameters or kinematics angles or even muscle strength and activity are considered. 

 Gait should be defined in a better way, especially how it is related to the different measures in terms of processes, even if there is anyway no real benefit demonstrated by stretching used as a unique therapy. Surprisingly, no article in children with cerebral palsy has been uncovered, while the clinical care of these children is mostly based on muscle stretching.

Are the rationale for, and objectives of, the Systematic Review clearly stated?

Yes

Is the statistical analysis and its interpretation appropriate?

Yes

Are sufficient details of the methods and analysis provided to allow replication by others?

Yes

Are the conclusions drawn adequately supported by the results presented in the review?

Partly

Reviewer Expertise:

motor control, biomechanics, neurosciences, nonlinear dynamics, clinical gait/movement analysis and gross motor function of children with cerebral palsy

I confirm that I have read this submission and believe that I have an appropriate level of expertise to confirm that it is of an acceptable scientific standard, however I have significant reservations, as outlined above.

# Response

**Published:** 2020-10-20

**Authors:** ARNAUD DELAFONTAINE

**Affiliations:** INRA Université Paris Sud, France

Reviewer 1 :

Dear Reviewer

We greatly you for scrutinizing our manuscript and for his relevant comments. We feel that the manuscript has improved.

Comment 1. In young adults, two studies by Johanson are reported. Is it not clear if both groups of young adults with limited range of motion were identical in both studies and had the same characteristics? If yes, merging the two studies should make the deal, if not please provide specification about the second group.The participants in both studies were different. In the study of Johanson et al. (2006), the participants were healthy adults with limited passive ankle-dorsiflexion range of motion (less than 8 degrees) **and** **a history of lower limb overuse injury**. In the study Johanson et al. (2009), the characteristics of the stretching program were the same as in Joahnson et al. (2006), but the participants did not have any history of lower limb overuse injury.

**Reply:** To make this difference clearer, precisions were added in the “included studies” and “results” sections:

Please see L158-163 Thus, 12 articles were ultimately included in this systematic review. Six studies evaluated the effects of stretching in healthy older adults ^23,51–55^, one in a frail elderly population ^56^, one study in an elderly population with stable symptomatic peripheral artery disease ^57^, one in stroke patients ^58^, one study in adults with limited ankle range of motion (less than 8 degrees) associated with a history of lower limb overuse injury ^59^, one study in healthy adults with limited ankle dorsiflexion  range of motion (less than 5 degrees) ^60^ and one in healthy young adults ^61^

Please see L285-288: “The study of Johanson et al. (2006) examined the effects of stretching on healthy adults with limited passive ankle-dorsiflexion range of motion (less than 8 degrees) and a history of lower limb overuse injury ^50^. Regarding the characteristics of the subjects, 19 subjects were included and the mean age was 30.3±9.8 years.”

Please see L302-306: “The study of Johanson et al. (2009) examined the effects of stretching on young adults with limited passive ankle-dorsiflexion range of motion (less than 5 degrees) ^51^. It is worth noticed that these participants were not the same than in the study of Johanson et al. (2006). In contrast,  the characteristics of the training programs were the same as in Johanson et al. (2006)”.

Comment 2. In the results and the discussion, grouping the types of parameters according to their role in the process of walking would add value to this manuscript. The effects, demonstrated or not, have not all the same meaning depending on whether functional parameters or kinematics angles or even muscle strength and activity are considered.

**Reply: **In the results and the discussion, we chose to group the participants by patient categories because differences in ages and health parameters may result in different training effects. However, in each patient category, each variable was considered separately in an organized way.

Comment 3. Gait should be defined in a better way, especially how it is related to the different measures in terms of processes, even if there is anyway no real benefit demonstrated by stretching used as a unique therapy.

**Reply:** We agree. We add a whole paragraph at the beginning of the introduction section to specify how gait can be related to the different variables seen in the review:

Please see L35-44: “Gait is the medical term used to describe the human whole body movement of walking ^1^. Gait involves internal and external forces that act on the body to move the center of mass (COM) across a given distance ^2^. It depends on many biomechanical features that can be observed during gait analysis such as center of mass shifts, joint range of motion, forces, muscle activity, joint moments, and joint powers ^3^. Spatiotemporal features (e.g. velocity, step length, stride length, step with, step variability) and kinematics parameters (range of motion) can be observed subjectively with functional evaluations by clinicians(e.g. the Tinetti test ^4^ or the timed up and go test ^5^), but, it can be further objectified with biomechanical analysis in a laboratory ^2^. Kinetics variables (the forces that cause the body to move) must be collected in a laboratory environment with force plates (e.g. ^6–9^ for recent studies that used this technic)”.

Comment 4. Surprisingly, no article in children with cerebral palsy has been uncovered, while the clinical care of these children is mostly based on muscle stretching.

**Reply:** We agree. However, no article in children with cerebral palsy fitted our inclusion criteria during the systematic search of the literature. We add a whole paragraph in a specific section “limitations of the study”:

Please see L397-406: Some patient categories were not included in the present review, although muscle stretching is commonly indicated in their clinical care to reduce spasticity ^82^. This is for example the case for children with cerebral palsy ^83^. In fact, we were able to identify studies in the literature focusing on the effects of stretching on gait in this population during the first phase of the present review, but the protocol of these studies combined stretching with another form of training (e.g. ^84,85^) or there was no control group (e.g. ^86^). These studies therefore did not fit with the inclusion criteria of the present systematic review and were consequently excluded. Now, it should be stressed that the effectiveness of static stretching to improve motor function in children with cerebral palsy is still controversial ^87^, although some authors showed that functional stretching exercises may be effective to improve gait ^86^. Further randomized controlled trials are needed to explore the impact of stretching on gait in this population.

Best regards 

Arnaud Delafontaine 

on behalf of all the authors
